# Exosomes Communicate Protective Messages during Oxidative Stress; Possible Role of Exosomal Shuttle RNA

**DOI:** 10.1371/journal.pone.0015353

**Published:** 2010-12-17

**Authors:** Maria Eldh, Karin Ekström, Hadi Valadi, Margareta Sjöstrand, Bob Olsson, Margareta Jernås, Jan Lötvall

**Affiliations:** 1 Krefting Research Centre, Dept. of Internal Medicine, Sahlgrenska Academy, University of Gothenburg, Gothenburg, Sweden; 2 Department of Rheumatology and Inflammation Research, Sahlgrenska Academy, University of Gothenburg, Gothenburg, Sweden; 3 Department of Internal Medicine, Sahlgrenska Academy, University of Gothenburg, Gothenburg, Sweden; Institut Pasteur, France

## Abstract

**Background:**

Exosomes are small extracellular nanovesicles of endocytic origin that mediate different signals between cells, by surface interactions and by shuttling functional RNA from one cell to another. Exosomes are released by many cells including mast cells, dendritic cells, macrophages, epithelial cells and tumour cells. Exosomes differ compared to their donor cells, not only in size, but also in their RNA, protein and lipid composition.

**Methodology/Principal Findings:**

In this study, we show that exosomes, released by mouse mast cells exposed to oxidative stress, differ in their mRNA content. Also, we show that these exosomes can influence the response of other cells to oxidative stress by providing recipient cells with a resistance against oxidative stress, observed as an attenuated loss of cell viability. Furthermore, Affymetrix microarray analysis revealed that the exosomal mRNA content not only differs between exosomes and donor cells, but also between exosomes derived from cells grown under different conditions; oxidative stress and normal conditions. Finally, we also show that exposure to UV-light affects the biological functions associated with exosomes released under oxidative stress.

**Conclusions/Significance:**

These results argue that the exosomal shuttle of RNA is involved in cell-to-cell communication, by influencing the response of recipient cells to an external stress stimulus.

## Introduction

Exosomes are 30-100 nm extracellular membrane vesicles of endocytic origin [1]-[3], which were first discovered in the early 1980's [1], [4]–[5]. Exosomes are released into the extracellular environment upon fusion of multivesicular bodies with the plasma membrane [1]–[2], [6]. They are secreted by most cells that have been examined so far, including mast cells [7]–[8], dendritic cells [9]–[10], B cells [6], T cells [11], tumour cells [12]–[13] and epithelial cells [14]. They have also been found in many biological fluids including plasma [15], urine [16], saliva [17], breast milk [18] and bronchoalveolar lavage fluid [19]. Exosomes were shown in the late 90's to have co-stimulatory functions in the immune system [6]. Furthermore, it has been shown that the exosome protein composition depends on the cellular source of the studied exosome [10], [20]. Regardless of origin, several common proteins are found in exosomes, including chaperones, cytoskeletal proteins and tetraspanins such as CD9, CD63 and CD81 [3], [8], [20]. We have previously shown that exosomes also contain a substantial amount of RNA that can be transferred from one cell to another [8]. The functions of exosomes are not yet fully understood, although antigen presentation [6], [21], induction of tolerance [22] and the transfer of genetic material [8] are the main proposed functions. The detailed mechanism of the interaction between exosomes and recipient cells are not fully understood, although experimentally supported hypotheses includes receptor-ligand interaction [6], [21], fusion with the plasma membrane [23] or internalization of the exosomes by the recipient cells by endocytosis [24]–[25] followed by uptake of functional RNA [8].

Reactive oxygen species (ROS), including hydrogen peroxide (H_2_O_2_), are continuously generated during cellular metabolism in cells living under aerobic conditions. If the ROS production exceeds the production of the cells antioxidant defence, an imbalance occurs resulting in oxidative stress, which is implicated in many diseases including cardiovascular disease [26], sleep apnoea [27], asthma [28]–[29] and COPD [28]. In higher doses, H_2_O_2_ is capable of inducing oxidative stress in experimental models [30]–[31], which can lead to different types of cell death [32]–[33]. In addition, low doses of H_2_O_2_ can induce tolerance of cells to a higher degree of oxidative stress [34]–[36]. Protection from oxidative stress has been shown to be regulated at the transcriptional level [37]–[39].

Since exosomes are produced and released by many cells, and have diverse functions in biological models [3], [40], we hypothesized that exosomes may mediate protective signals in processes of oxidative stress. Thus, we suggest that exosomes released by cells exposed to oxidative stress can mediate a signal to another cell, making the recipient cell more tolerant to oxidative processes and subsequent cell death. We further hypothesized that any tolerising effect can be mediated by the exosomal shuttle of RNA, as we have previously shown that exosomes can deliver functional RNA from one cell to another [8]. To test these hypotheses, we used a mouse mast cell line (MC/9) that we exposed to H_2_O_2_, as a model of oxidative stress.

## Results

### Exosomes alter the ability of cells to handle oxidative stress

**Figure 1:**
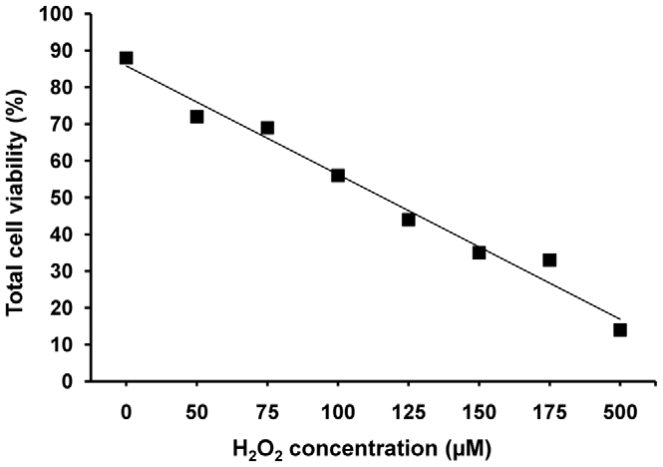
Oxidative stress induced by H_2_O_2_ results in a dose related loss of viability. Dose response relationship between viability of cultured MC/9 cells (%) and concentration of H_2_O_2_ (50 µM-500 µM) for 24 h. The dose versus viability correlation coefficient was 0.86.

**Figure 2:**
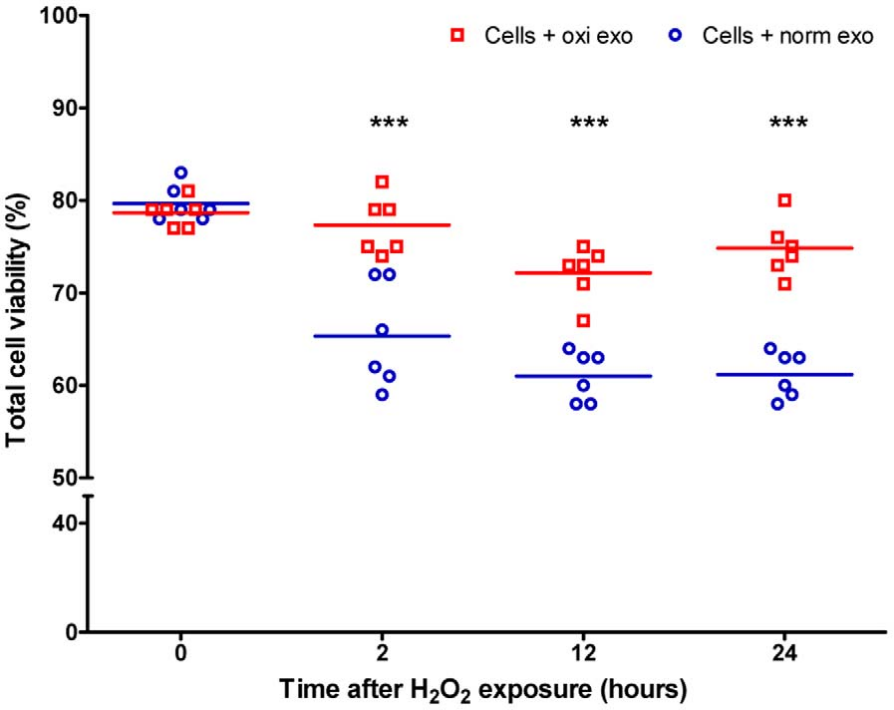
MC/9 cells pre-treated with exosomes released under oxidative stress obtain a resistance to oxidative stress. Time course of viability of MC/9 cells (n = 6) (%) after exposure to oxidative stress (H_2_O_2_ 125 µM) when pre-treated with exosomes derived from other MC/9 cells that were either exposed (oxi exo) or not exposed (norm exo) to H_2_O_2_ at the same concentration. Treatment of MC/9 cells with exosomes released under oxidative stress increased viability with approximately 15-20% at different time points after the initiation of H_2_O_2_ exposure. ***p<0.001.

It is known that oxidative stress induced by H_2_O_2_ induces loss of cell viability *in vitro* [33]. Depending on cell type, the dose of H_2_O_2_ needed to induce loss of viability differs. A dose-response evaluation was performed, after which we concluded that the concentration of 125 µM was optimal for our protocol as this dose caused the death of about 50% of the cells (Figure 1). It has previously been documented that cells pre-treated with a low H_2_O_2_ dose develop a resistance to higher doses of H_2_O_2_ and consequently to stress [34]–[36]. To determine whether exosomes released under oxidative stress can mediate a similar tolerising effect, we harvested exosomes from MC/9 cells exposed to H_2_O_2_ or vehicle for 24 h. These exosomes were then added to untreated cultures of other MC/9 cells for 3 h, after which the recipient cells were exposed to oxidative stress at the same concentration. Recipient cell viability was examined at 0, 2, 12 and 24 h after H_2_O_2_ exposure, by trypan blue dye exclusion. Cells pre-treated with exosomes harvested from conditions of oxidative stress, were shown to have a higher viability at the 0, 2, 12 and 24 h time points, compared to cells pre-treated with exosomes harvested from normal conditions (Figure 2).

### Exposure of cells to oxidative stress increase the relative amount of oxidized proteins in cells, but not in exosomes

**Figure 3:**
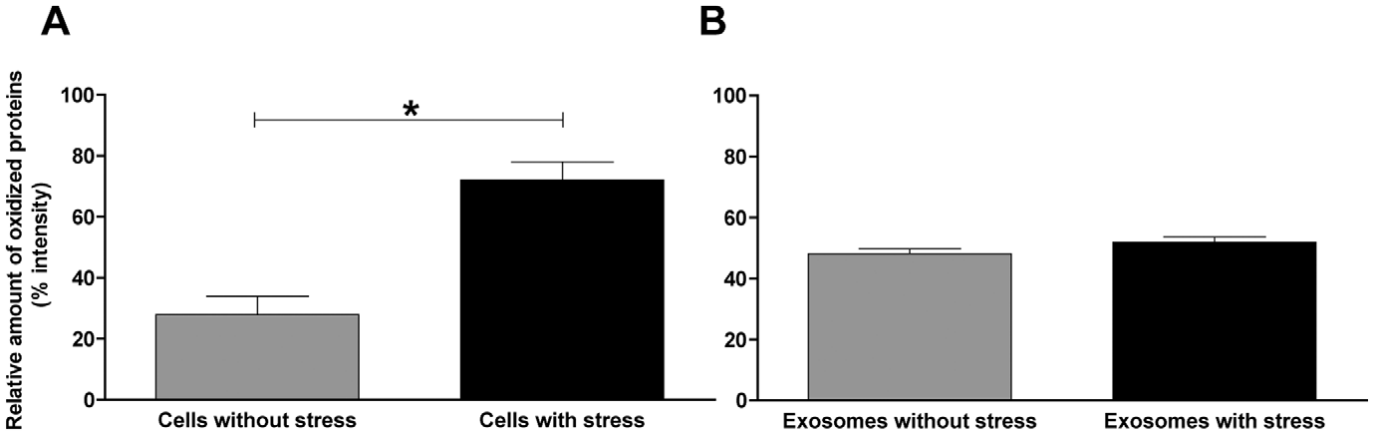
Cells exposed to H_2_O_2_ show an increase in oxidized proteins, whereas exosomes do not. Oxidized proteins (% intensity) in MC/9 cells (a) and their released exosomes (b) after exposure to vehicle or H_2_O_2_ (125 µM) for 24 h (n = 5). Oxidative stress significantly increased the relative amount of oxidized proteins in cells, but did not significantly affect the relative amount of oxidized proteins in exosomes. *p<0.05.

After showing that exosomes harvested from cells cultured under oxidative stress were capable of mediating resistance to oxidative stress, we next compared the degree of oxidization of cellular and exosomal proteins. This was performed by studying the carbonyl groups, introduced by the H_2_O_2_ exposure, using a protein oxidation detection kit with a specific antibody targeting these carbonyl groups. We could, as previously shown [41], see an increase of oxidized proteins in cells exposed to H_2_O_2_ (Figure 3a). However, the proteins in exosomes derived from cells exposed to H_2_O_2_ did not express any change in the degree of oxidization (Figure 3b).

### Microarray analysis reveals that exosomes from different conditions contain different mRNA expression

**Figure 4:**
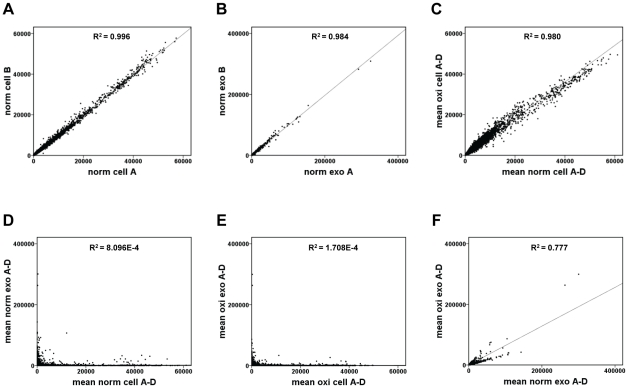
Scatter plots of relationships between mRNA signals in MC/9 cells and exosomes. a) Reproducibility comparison of mRNA signals between two different cell cultures of MC/9 cells (sample norm cell A and norm cell B) under normal conditions (norm). b) Reproducibility of comparison of mRNA signals between exosomes derived from two different cell cultures of MC/9 cells (sample norm exo A and norm exo B) under normal conditions (norm). c) Relationship between mean mRNA signals between MC/9 cells (samples norm cell A–D and oxi cell A–D) that have been exposed to vehicle (norm) or 125 µM H_2_O_2_ (oxi) for 24 h. d) Relationship between mean mRNA signals in MC/9 cells and their released exosomes (samples norm cell A–D and norm exo A–D) under normal conditions (norm). e) Relationship between mean mRNA signals in MC/9 cells and their released exosomes (samples oxi cell A–D and oxi exo A–D) after H_2_O_2_ exposure for 24 h (125 µM) (oxi). f) Relationship between mean mRNA signals in exosomes released from MC/9 cells (samples norm exo A–D and oxi exo A–D) after exposure to vehicle (norm) or H_2_O_2_ (125 µM) (oxi) for 24 h.

In our previous publication, we showed that exosomes contain not only protein, but also mRNA and microRNA [8]. Importantly, we also showed that the mRNA is functional and can be shuttled between cells. As we have shown that exosomes harvested from oxidative stress conditions affect the recipient cells extensively, we examined whether the exosomal mRNA content had changed. This was evaluated by isolation of RNA followed by Affymetrix microarray analysis. This analysis was performed on RNA from both the exosomes and their donor cells. The Affymetrix microarray analysis confirmed our previously published results [8], that there is no correlation between cellular mRNA and the exosomal mRNA indicating a difference in mRNA content (Figure 4d). In addition, this lack of correlation was also seen between donor cell and exosomal mRNA under oxidative stress (Figure 4e). Importantly, a difference in mRNA content was observed between exosomes harvested from the different conditions (Figure 4f). Furthermore, the results also showed a slight difference in gene expression in cells cultured under normal conditions compared to oxidative stress (Figure 4c).

**Figure 5:**
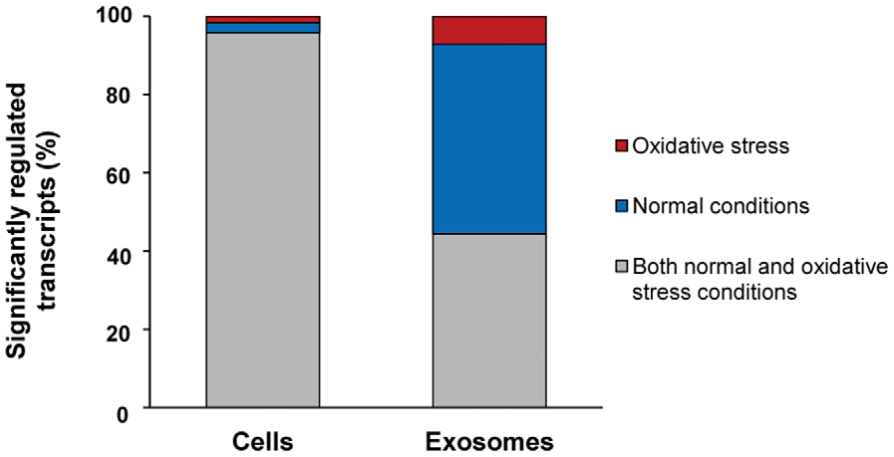
Relationship between significantly changed transcripts in MC/9 cells and exosomes, from normal and oxidative stress conditions. Significantly regulated transcripts found in cells and exosomes (n = 4, all present) from both normal conditions and from H_2_O_2_ (125 µM, 24 h) induced oxidative stress. The majority of the cellular transcripts are the same in both cells grown under normal conditions and in cells grown under oxidative stress, as shown by the grey field, and only a small percentage of the transcripts change depending on the condition. The blue field shows the transcripts that are only expressed in cells grown under normal conditions and the red field shows transcripts only expressed in cells grown under oxidative stress conditions. However, the significantly regulated transcripts in exosomes change vastly depending on the condition compared to the cells.

Interestingly, the relationship between significantly regulated transcripts found in exosomes from normal conditions and from oxidative stress were shown to change substantially in exosomes, although in cells this relationship between the two conditions were similar (Figure 5).

The top 20 up- and down-regulated genes in exosomes harvested from cells cultured under oxidative stress are shown in Table 1 and 2 respectively.

**Table 1:** Induced genes in exosomes released under oxidative stress.

Gene symbol/Gene name	Mean signal norm exo	Mean signal oxi exo	Fold change	p-value
[Vsig1] V-set and immunoglobulin domain containing 1	200	663	3.3	1.66E-03
[Top1] topoisomerase (DNA) I	540	1402	2.6	4.63E-02
[Ccbp2] chemokine binding protein 2	356	897	2.5	3.32E-02
[0610010K06Rik] RIKEN cDNA 0610010K06 gene	383	966	2.5	1.32E-02
[Krit1] KRIT1, ankyrin repeat containing	325	811	2.5	2.41E-02
[D230019N24Rik] RIKEN cDNA D230019N24 gene	426	1015	2.4	5.42E-03
[Amy2a1] amylase 2a1, pancreatic	349	827	2.4	7.77E-04
[Lba1] lupus brain antigen 1	532	1230	2.3	3.92E-02
[Zfp385c] zinc finger protein 385C	585	1349	2.3	1.73E-02
[2700057C20Rik] RIKEN cDNA 2700057C20 gene	528	1213	2.3	2.52E-03
[Ptar1] protein prenyltransferase alpha subunit repeat containing 1	688	1557	2.3	2.55E-02
[Smad3] MAD homolog 3 (Drosophila)	593	1339	2.3	2.35E-02
[2810002D19Rik] RIKEN cDNA 2810002D19 gene	239	530	2.2	1.10E-02
[Phf6] PHD finger protein 6	527	1154	2.2	1.06E-02
[Hsd17b11] hydroxysteroid (17-beta) dehydrogenase 11	386	813	2.1	3.18E-02
[6720457D02Rik] RIKEN cDNA 6720457D02 gene	963	2023	2.1	4.95E-02
[Yipf7] Yip1 domain family, member 7	705	1463	2.1	1.59E-02
[Mep1a] meprin 1 alpha	375	765	2.0	3.87E-02
[Sox15] SRY-box containing gene 15	318	648	2.0	4.73E-02
[4930473M17Rik] RIKEN cDNA 4930473M17 gene	421	845	2.0	3.03E-03

This table shows the 20 most induced mRNA transcripts in exosomes derived from MC/9 cells exposed to oxidative stress (H_2_O_2_, 125 µM for 24 h, oxi exo) compared to exosomal mRNA transcripts after exposure of cells to vehicle (norm exo). A fold-change of e.g. 2 indicated that the gene is 2 fold up-regulated in the exosomes derived from cells exposed to oxidative stress.

**Table 2:** Repressed genes in exosomes released under oxidative stress.

Gene symbol/Gene name	Mean signal norm exo	Mean signal oxi exo	Fold change	p-value
[Ctnna1] catenin (cadherin associated protein), alpha 1	1417	176	−8.0	1.83E-02
[Pigq] phosphatidylinositol glycan anchor biosynthesis, class Q	1661	212	−7.8	4.15E-02
[Cct2] chaperonin containing Tcp1, subunit 2 (beta)	2094	278	−7.5	9.33E-04
[Rfc4] replication factor C (activator 1) 4	1115	149	−7.5	5.48E-03
[Gnas] GNAS (guanine nucleotide binding protein, alpha stimulating) complex locus	2193	324	−6.8	5.96E-03
[Ttc3] tetratricopeptide repeat domain 3	1701	253	−6.7	4.35E-02
[Laptm5] lysosomal-associated protein transmembrane 5	15814	2461	−6.4	9.80E-03
[Gabarapl1] gamma-aminobutyric acid (GABA) A receptor-associated protein-like 1	1180	188	−6.3	1.61E-03
[Ipo4] importin 4	1706	276	−6.2	2.10E-02
[Dnpep] aspartyl aminopeptidase	5271	871	−6.1	8.76E-03
[Lmna] lamin A	1918	329	−5.8	1.52E-02
[Ssr3] signal sequence receptor, gamma	5227	912	−5.7	1.96E-02
[Qars] glutaminyl-tRNA synthetase	1905	341	−5.6	1.54E-04
[Gsn] gelsolin	4203	811	−5.2	1.53E-02
[Arap3] ArfGAP with RhoGAP domain, ankyrin repeat and PH domain 3	2416	470	−5.1	6.95E-03
[Med22] mediator complex subunit 22	3454	679	−5.1	3.06E-02
[Csnk1d] casein kinase 1, delta	1383	275	−5.0	6.96E-03
[Coro7] coronin 7	2339	465	−5.0	1.18E-02
[Lasp1] LIM and SH3 protein 1	3478	698	−5.0	5.03E-05
[Ric8] resistance to inhibitors of cholinesterase 8 homolog (C. elegans)	1956	404	−4.8	4.88E-02

This table shows the 20 most repressed mRNA transcripts in exosomes derived from MC/9 cells exposed to oxidative stress (H_2_O_2_, 125 µM for 24 h, oxi exo) compared to exosomal mRNA transcripts after exposure of cells to vehicle (norm exo). A fold-change of e.g. 2 indicated that the gene is 2 fold down-regulated in the exosomes derived from cells exposed to oxidative stress.

### UV- light eliminates the protective effect of exosomes against oxidative stress

**Figure 6:**
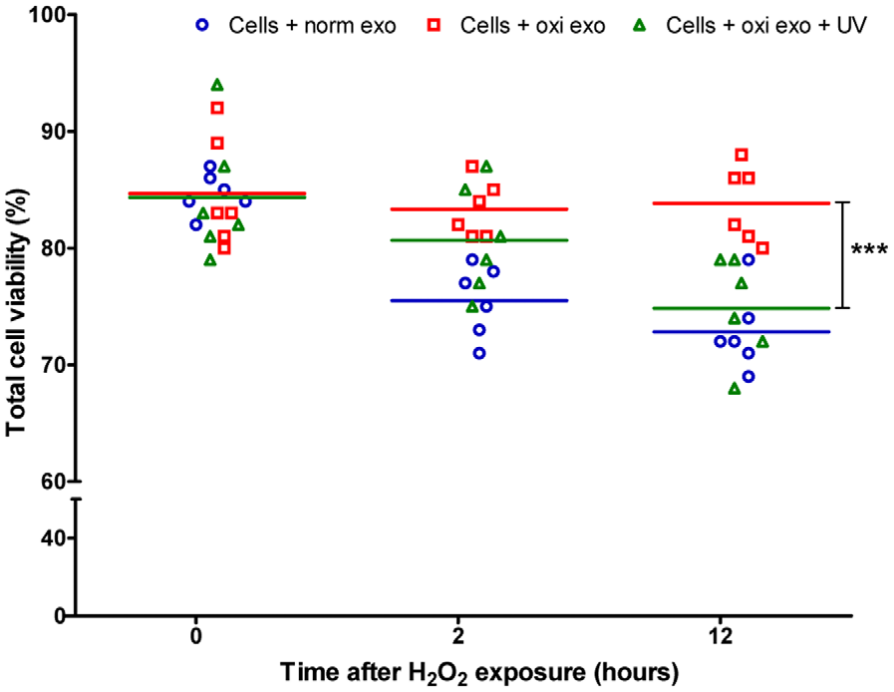
UV-light removes the conditioning effect of exosomes released under oxidative stress. Effect of UV-light exposure on the conditioning effect of exosomes on recipient cell tolerability to H_2_O_2_ exposure. Illustrated by viability of MC/9 cells (n = 4) (%) after exposure to oxidative stress (H_2_O_2_ 125 µM) when pre-treated with exosomes derived from other MC/9 cells that were exposed to H_2_O_2_ (oxi exo) and treated or not treated with UV-light (254 nm) for 1 h at low temperature (0–4°C). Treatment of exosomes derived from cells grown under oxidative stress with UV-light totally eliminated the protective effect of these exosomes on cell viability at 12 h. ***p<0.001.

Since the mRNA content of exosomes differs substantially in exosomes released under oxidative stress compared to exosomes released under normal conditions, and as exosomes released under oxidative stress can induce a resistance against oxidative stress in recipient cells, we hypothesized that the conditioning effect could be mediated by the RNA content in exosomes. To test this hypothesis, exosomes harvested from oxidative stress were exposed to UV-light (254 nm) for 1 h, as UV-light inactivates RNA functions [42]–[43]. As controls, exosomes from both normal and stressed conditions were treated in parallel, but without exposure to UV-light. After the UV-light exposure, the exosomes were added to untreated cultures of recipient cells which were then exposed to oxidative stress, as in the previous experiments, and any influence on cell viability was determined at 0, 2 and 12 h. The results revealed that exosomes exposed to UV-light lost their protective effect on the viability of recipient cells exposed to oxidative stress at the 12 h time point (Figure 6).

## Discussion

This study shows that exosomes, released from mast cells exposed to oxidative stress, have the capacity to communicate a protective signal to recipient cells exposed to subsequent oxidative stress, resulting in reduced cell death. The mRNA content of exosomes produced under oxidative stress differs extensively from both the mRNA in the donor cell and in the exosomes produced by cells cultured under normal conditions. UV-light exposure, which damages nucleic acids [42]–[44] and proteins [45], eliminate the exosomal protective signal, which may suggest that the exosomal shuttle of RNA at least partly mediate the observed effect.

Exosomes harvested from different cells under different situations have been shown to mediate a multitude of biological effects, including antigen presentation [6], [21], induction of apoptosis [46], and promotion of cancer cell growth [47] as a few examples. The current study adds to the list of biological functions of exosomes, proving that exosomes produced during oxidative stress mediate protective signals to the same stress in other cells. Thus, we observed that exosomes, released by cells grown under oxidative stress, reduce cell death. Consequently, we show that the oxidative stress alters the biological function of exosomes released from mast cells, which further argues that these vesicles can communicate important regulatory signals from one cell to another.

As shown in previous studies, we confirm that exposure of mast cells to H_2_O_2_ results in reduced cell viability *in vitro* [33]. To study the effects of exosomes, we were careful to choose a dose of H_2_O_2_ that resulted in a moderate degree of cell death, to be able to study any up or down regulating effects of exosomes. It is well known that oxidative stress can lead to various cell damage such as lipid peroxidation, nucleic acids oxidation and protein oxidation [41], [48]–[49]. The results of protein oxidation by ROS are many, including cleavage of peptide bonds, cross-linkage reactions and generation of carbonyl derivates [49]. Interestingly, the dose of H_2_O_2_ to induce oxidative stress resulted in an increased relative amount of introduced carbonyl groups in the proteins of exposed cells, but not in the proteins of exosomes that they released. Thus, the cells seem to be extensively affected themselves by the oxidization process, unlike the exosomes. We suggest that the cells may actively protect the exosomes from containing damaged proteins by specifically packaging the exosomes with undamaged proteins. This data also argues that the conditioning signal mediated by exosomes released during oxidative stress, is not mediated by oxidized exosomal proteins *per se*.

In previous work, we have shown that the RNA content in exosomes differs extensively from the donor cell's RNA [8]. In the current study, we hypothesized that the exosomal RNA content changes, and that this change is not only dependent on the cell origin but also on the condition under which they have been produced and released under, in this case normal conditions and oxidative stress. Indeed, the Affymetrix microarray analysis show substantial differences in mRNA gene expression in exosomes compared to their donor cells, both from cells with and without exposure to oxidative stress. Also, the exosomal mRNA content substantially differed in exosomes harvested from cells grown under the different conditions, arguing that the RNA content in exosomes is closely regulated depending on a cell's biological state or function. This result confirms our previous conclusion that the mRNA content in the exosomes is not a random sample of the cellular mRNA [8], as it differed substantially from the donor cell mRNA regardless of the cell culture conditions.

Since the exosomal RNA content changed extensively under conditions of oxidative stress and because we have previously shown that the exosomal shuttle of mRNA can result in translation of that mRNA in the recipient cell [8], we hypothesized that the protective effect of the exosomes released under these conditions is at least partly mediated by exosomal shuttling of RNA to recipient cells. To reduce the functionality of the RNA in the exosomes, we exposed the exosome fraction to UV-C radiation, as this treatment is known to have a damaging effect on nucleic acids [42]–[44], [50]. After this treatment, we found that the exosomes harvested from oxidative stress lose their ability to protect recipient cells from oxidative stress. These results therefore argue that the conditioning signals at least partially may be with the exosomal RNA content, and further supports the notion that the exosomal RNA indeed has regulatory functions in situations of biological importance. However, as it is also known that proteins can be damaged by UV-light [45], a biological role of exosomal proteins in this experiment cannot be excluded.

It is clear that exosomes harvested from different cells and under different conditions have vastly diverse effects in different cell systems. This suggests that exosomes can have a multitude of effects *in vivo*, depending on how and where they were produced. Many studies suggest that the core protein content of exosomes in fact are conserved [3], [20], whereas the RNA content in exosomes, according to our current findings, can change extensively under different conditions. It is therefore possible that many of the diverse functions of exosomes reported in different studies are in fact mediated by different RNA signals that are shuttled between cells by exosomes. The current study therefore further emphasizes the putative biological regulatory importance of the shuttling of RNA between cells by exosomes.

In conclusion, in this study we have shown that exosomes that are produced by cells exposed to oxidative stress have the ability to induce tolerance to oxidative stress in another cell. This effect is associated with changed exosomal mRNA content that can be attenuated by reduced RNA activity through exposure to UV-light. This shows, for the first time, that the exosomal shuttle of RNA can fundamentally change the biological function of a recipient cell. When functions of exosomes are pursued, the role of their RNA content should be carefully considered.

## Materials and Methods

### MC/9 cell culture, oxidative stress treatment and exosome isolation

MC/9 cells (ATCC, Manassas, VA) were cultured in Dulbecco's Modified Eagle's Medium, 10% fetal bovine serum (FBS), 100 µg/ml penicillin-streptomycin, 2 mM L-glutamine, 0.05 mM 2-mercaptoethanol (all from Sigma-Aldrich, St Louis, MO, USA) and 10% Rat T-Stim (BD Biosciences, Erembodegem, Belgium), at 37°C and 5% CO_2_. The FBS and Rat T-Stim contain exosomes. To remove these exosomes, FBS and Rat T-Stim were ultracentrifuged at 120,000 g for 90 min, 4°C (Ti45 rotor, Beckman Coulter, Brea, CA, USA). To induce oxidative stress, cells were exposed to 125 µM H_2_O_2_ (Sigma-Aldrich) for 24 h under culture conditions. For isolation of exosomes, MC/9 cell suspension was centrifuged for 10 min at 300 g, to pellet the cells, and the exosomes were prepared from the supernatant. The exosomes were purified by ultracentrifugation in a Beckman Ultracentrifuge (rotor Ti45). First, the debris and organelles of the culture were precipitated by centrifugation (20 min, 16,500 g, 4°C) and the supernatant was filtered through a 0.2 µm filter, to remove any molecules larger than 200 nm. The exosomes were then pelleted by an ultracentrifugation at 120,000 g, 70 min, 4°C.

### Total RNA purification and analysis

Total RNA was extracted from cells and exosomes (n = 4) by Trizol® extraction methodology (Invitrogen, Paisley, UK) according to the manufacturer's protocol. In short, samples were homogenized and RNA integrity maintained by Trizol®. RNA, DNA, and proteins were then separated into different phases. After centrifugation, the RNA was collected from the aqueous phase, precipitated, washed and resuspended in RNase free water. The Mouse Genome 430A 2.0 microarray (Affymetrix, Santa Clara, CA, USA) was performed by SweGene (www.swegene.org/) according to Affymetrix microarray DNA chip analysis (Affymetrix). Gene expression profiles were analyzed using the MAS5.0 software (Affymetrix).

### Accession Number

The microarray data have been deposited in NCBI's Gene Expression Omnibus (GEO). Details can be found at http://www.ncbi.nlm.nih.gov/geo (the GEO accession number is: GSE24886).

### Transfer experiment and cell viability analysis

All exosomes were isolated (n = 6) from MC/9 donor cells exposed to H_2_O_2_ (125 µM) or vehicle (complete medium) for 24 h and redissolved in complete medium. All of the exosomes collected from the supernatant from the donor cell cultures were added to the MC/9 recipient cells in the ratio of 1.7∶1. This approach was taken to ensure that all exosomes and their content were transferred, which would better reflect the true biological state as opposed to a small subset. The recipient cells and exosomes were then incubated for 3 h under normal culture conditions. The recipient cells were subsequently challenged with H_2_O_2_ (125 µM) and harvested after 0, 2, 12 and 24 h. The cell viability was assessed by using the trypan blue dye exclusion method.

### Detection of oxidized proteins

The total protein was extracted from cells and exosomes (n = 5) using modified RIPA buffer [51] and sonication. Cell debris was removed by centrifugation. Detection and quantification of oxidized proteins was performed using the OxyBlot™oxidized protein detection kit (Millipore, Billeria, MA, USA) according to the manufacturer's recommendations. In brief, the protein carbonyl groups, which are a consequence of the oxidative stress modification, were derivatized. Equal amounts of protein (15–20 µg) were then separated on polyacrylamide gels, transferred onto nitrocellulose membranes (Bio-Rad, Hercules, CA, USA) and blotted using antibodies specific to the OxyBlot™ kit. Enhanced chemiluminescence (GE Healthcare, Uppsala, Sweden) and Quantity One® software (Bio-Rad) was then used for visualisation and relative quantification.

### Exposure of exosomes to UV-light and subsequent transfer

All exosomes were isolated (n = 6) from MC/9 donor cells exposed to H_2_O_2_ (125 µM) or vehicle (complete medium) for 24 h and resuspended in PBS. Exosomes isolated from cells exposed to H_2_O_2_ were then subjected to UV-light (254 nm) for 1 h at 0–4°C. As controls, exosomes released by cells exposed to H_2_O_2_ or vehicle, not subjected to UV-light, were kept at 4°C for 1 h. The exosomes were then added to MC/9 recipient cells in the ratio of 1.7∶1 between donor cells and recipient cells and incubated for 3 h under normal culture conditions. The recipient cells were subsequently challenged with H_2_O_2_ (125 µM) and harvested after 0, 2 and 12 h. The cell viability was assessed by using the trypan blue dye exclusion method.

### Statistical analysis

Where appropriate, data are expressed as mean ±SEM. Statistical analysis was performed by one-way ANOVA test when comparing more than two groups and paired t-test (two tailed) analyses were used when comparing two conditions (SPSS for Windows® version 17.0). Differences in gene expression between normal conditions and oxidative stress were assessed with paired t-test (two tailed). A probability less than 0.05 was accepted as statistically significant.
